# Telework-related risk factors for musculoskeletal disorders

**DOI:** 10.3389/fpubh.2023.1155745

**Published:** 2023-07-03

**Authors:** Marina Milaković, Helena Koren, Karmen Bradvica-Kelava, Marija Bubaš, Josipa Nakić, Pavle Jeličić, Lovro Bucić, Barbara Bekavac, Jelena Čvrljak, Magdalena Capak

**Affiliations:** ^1^Division for Occupational Health, Croatian Institute of Public Health, Zagreb, Croatia; ^2^Faculty of Kinesiology, University of Zagreb, Zagreb, Croatia; ^3^Division for Environmental Health, Croatian Institute of Public Health, Zagreb, Croatia; ^4^Andrija Stampar Teaching Institute of Public Health, Zagreb, Croatia; ^5^Department of History, Croatian Catholic University, Zagreb, Croatia

**Keywords:** telework, musculoskeletal disorders, ergonomic risks, psychosocial risks, COVID-19, pandemic

## Abstract

Telework has become considerably more common during the ongoing pandemic. Although working remotely may have numerous advantages, negative impacts on workers’ health and safety should also be considered. Telework is a major contributor to the development or aggravation of work-related musculoskeletal disorders where unsuited workstation ergonomics, sedentary behavior, as well as psychosocial and organizational factors play a role. This paper aims to identify telework-related risks and their impact on musculoskeletal health as well as provide recommendations that may be useful in constructing future preventive measures. A comprehensive literature search regarding the topic has been performed. Teleworkers experience musculoskeletal pain and discomfort mostly in low back area, neck, shoulders, arms, and hands. Poor ergonomic solutions when it comes to workstation design resulting in prolonged sitting in non-neutral positions contribute to the development and aggravation of musculoskeletal disorders in teleworkers. Working with inadequately placed screens and laptops and sitting in maladjusted seats without usual functionalities and ergonomic support is associated with musculoskeletal pain and discomfort. Extended working hours with fewer rest periods to meet increased work demands, social isolation, and lack of support from work colleagues and superiors as well as blurred work-home boundaries and omnipresence of work are commonly stated psychosocial and organizational factors associated with musculoskeletal disorders. Environmental factors such as poor lighting and glare, inadequate room temperature, and ventilation or noise, are frequently overlooked remote workstation risk factors. For a certain part of workers, telework will remain a common way of work in the post-pandemic period. Therefore, it is essential to identify telework-related risk factors for musculoskeletal disorders and address them with timely preventive measures tailored to each remote workstation’s risks and individual workers’ needs.

## Introduction

Computer work has previously been associated with poor musculoskeletal health where ergonomic (1), psychosocial (2), and individual (3) factors seem to play a role. Prolonged static posture and repetitive movements along with mental and visual strain arising from processing information and prolonged screen time are commonly associated with musculoskeletal health in office workers (1). Musculoskeletal disorders (MSDs) affect up to 72% of office workers who commonly experience neck, low back, shoulder, elbow, and wrist pain (4).

Research suggests that remote work may furtherly exacerbate MSDs in workers switching to remote working. Studies have shown that up to 61% of workers who transitioned to telework experience aggravation of musculoskeletal pain (5) often of moderate to severe intensity (6). When compared to office workers, teleworkers have an increased risk of pain in all body areas as well as an increased risk for pain severity (7). Commonly present increased working demands (5) and long working hours (8) with less frequent breaks (9) lead to prolonged exposure to computer-related risks while simultaneously adding new ones arising from characteristics of telework. A conceptual model describing factors, moderators, and mediators arising from job characteristics, remote work environment as well as from individual differences that may influence teleworkers’ health and well-being while teleworking has been proposed by Beckel and Fisher (10). It has been accentuated that frequently present poor ergonomic solutions when it comes to workstation design and equipment in remote setting result in prolonged static load in awkward positions with negative affect on musculoskeletal health (11). Often overlooked environmental factors such as poor lighting and glare, inadequate room temperature, and ventilation are workstation factors affecting body posture with significant association with MSDs (12). Most commonly reported psychosocial concerns arising from telework with a potential role in the onset or aggravation of MSDs include frequent interruptions due to household noise as well as a work-family imbalance caused by the omnipresence of work (13).

Before the pandemic, telework has shown to be prevalent in ICT-driven working sectors and encouraged as a mean to increase work productivity and quality, decrease a company’s costs and improve employees’ time management and quality of life (14, 15). The COVID-19 pandemic prompted the implementation of flexible work arrangements by imposing the need for social distancing to decrease person–to–person transmission of SARS-CoV-2, resulting in a significant increase in the number of workers that started to engage in flexible working arrangements. Research suggests that only 11% of workers in the European Union telecommuted in the pre-pandemic period with tripling percentages since the start of the pandemic (16). For some employees, telework has become a new and unknown way of working for which they were not adequately prepared, while in others telework evolved from part-to full–time working arrangement. Pandemic-related confinement led to a sudden transition to remote working, often with other household members, causing workers to perform their job in an unsuited setting with different risks that may potentially harm musculoskeletal health. Additionally, pandemic-related stress itself arising from fear of contagion and health deterioration as well as fear of financial problems may have an additional effect of pain/discomfort experienced in workers with pre-existing MSDs (17).

Considering the detrimental effect of musculoskeletal pain and disability on workers’ and organizational well-being, understanding factors arising from telework that may contribute to the onset or aggravation of MSDs that could be a potential target for preventive interventions is of great importance. Therefore, this mini-review aims to identify telework-related factors associated with the onset or aggravation of MSDs in teleworkers as well as provide recommendations that may be useful in constructing future preventive measures.

## Materials and methods

The MEDLINE/PubMed database was searched using MESH terms “teleworking” and “musculoskeletal pain.” Due to a limited number of articles (*N* = 3), the search was broadened using non-MESH terms ((“teleworking”[MeSH Terms] OR “teleworking”[All Fields] OR “telework”[All Fields]) AND musculoskeletal [All Fields]). Two authors independently reviewed available articles and decided on suitability for the current mini-review. References of selected articles were also reviewed to ensure the complete inclusion of relevant research. Only original research papers examining the association of telework conditions with musculoskeletal discomfort/pain were considered for inclusion.

## Results

The MEDLINE/PubMed search revealed 272 papers. Upon exclusion of papers unrelated to telework (*N* = 163), we furtherly excluded papers unrelated to musculoskeletal pain/discomfort (*N* = 75) as well as secondary publications, recommendations, and theoretical research (*N* = 6). Of the remaining 28 papers examining musculoskeletal pain/discomfort in teleworkers, 11 did not examine the association between telework working conditions and musculoskeletal discomfort. Finally, 17 research articles were included in the present mini review with one additional article obtained from references of included articles, resulting in 18 original research articles in total. Identified telework-related factors that showed association with the onset/aggravation of MSDs in teleworkers are shown in Table 1.

**Table 1:** Factors associated with MSDs in teleworkers.

References	Factors with significant correlation/association with MSD
Gosain et al. (11)	WH, dedicated workspace, psychological stress, breaks, eye strain, PA, gender
Yoshimoto et al. (17)	TW experience, psychological stress, PA
Snodgrass et al. (18)	Non-ergonomic equipment, postures
Radulović et al. (19)	WH, interruptions, non-ergonomic equipment, breaks, age
Du et al. (20)	WH, non-ergonomic equipment, gender
Garcia et al. (21)	TW frequency, living with more than one person, non-ergonomic equipment, prolonged sitting, lighting
El Kadri and Lucca (22)	Dedicated workspace
McAllister et al. (23)	Non-ergonomic equipment, perceived discomfort, ergonomic training
Matsugaki et al. (24)	TW frequency, dedicated workspace, spacious desk, lighting, air quality
Matsugaki et al. (25)	TW frequency, dedicated workspace, spacious desk, lighting, air quality
Minoura et al. (26)	TW experience, living with children, psychiatric disorders, cancer, smokers
Wütschert et al. (27)	Perceived privacy, relaxation
Gupta et al. (28)	WH, pre-existing MSD, sedentary time, gender
Oakman et al. (29)	Quantitative demands, work–family conflict, workstation comfort, gender
Rodríguez-Nogueira et al. (30)	Pre-existing MSD, gender
Tezuka et al. (31)	TW frequency
Dannecker et al. (32)	Self-rated health
Houle et al. (33)	Pre-existing MSD

TW, telework; MSD, musculoskeletal disorders; PA, Physical activity; WH, working hours.

## Discussion

The current mini review focused on telework-related factors associated with the onset or aggravation of MSDs in teleworkers. Factors arising from poor ergonomic solutions when it comes to workstation design, poor environmental conditions as well from increased workload with fewer breaks are most commonly reported. High job demands with frequent distractions as well as blurred work-family boundaries seem to be the most frequently observed psychosocial factors associated with MSDs in teleworkers.

Available research suggests that poor ergonomic workstation design is more prevalent in flexible ways of work (18) compared to office work. Teleworkers seem to frequently substitute office desks with dining, kitchen, or children’s desks and chairs (18–20) and may commonly be found working from a couch or bed in awkward and constraining postures. Additionally, they are more inclined to substitute traditional work setups consisting of desk computers, keyboards, and a mouse with other types of information and communication technology such as laptops, tablets, and phones (18, 21) with an additional negative impact on body postures (34). Prolonged static loading in awkward postures, along with prolonged sitting bouts and repetitive movement characteristic of computer work, may additionally negatively affect musculoskeletal health. Several selected studies (18–21) associated poorly designed ergonomic furniture with reported musculoskeletal pain/discomfort; however, due to the cross-sectional design of the studies causality cannot be determined. Snodgrass et al. (18) furtherly investigated differences in workstation settings and sitting postures in computer workers before and during the COVID-19 pandemic with results showing a decrease in good and an increase in poor sitting postures during confinement. Reason for such findings may be the limited availability of working equipment in the remote setting during the pandemic, particularly in shared households. As mentioned before, the pandemic has caused a sudden transition to new ways of work for which employees may not be adequately prepared. It is possible that multiple members of a household suddenly transitioned to working or schooling from home limiting working space and equipment, and with the need for mutual usage and sharing. El Kadri and Lucca (22) observed differences in ergonomic risks concerning previous experience in telework suggesting higher ergonomic risks in workers starting telework during the pandemic, with unpreparedness as a possible explanation. However, considering that research has been conducted one and a half year after the start of the pandemic, authors suggest that, along with the novelty of the pandemic, organizational and individual lack of initiative for evaluation and adaptation of employees’ working conditions may contribute to poorer working conditions in inexperienced workers (22). Studies have previously pointed out the lack of organizational support for teleworkers when it comes to providing ergonomic equipment as well as support and guidance in installation and usage (35). Research suggests that besides providing ergonomic furniture and equipment, education on how to properly set up workstations may be of as much importance. However, studies have also shown that almost 60% of teleworkers do not receive basic guidance on setting up their workplaces (14). By showing the interaction of reported MSDs with a model consisting of non-ergonomic furniture, perceived discomfort, and ergonomic training, McAllister et al. (23) additionally pointed out the importance of education and ergonomic training in setting up a remote workstation in the prevention of MSDs.

Perceived discomfort may only partially be related to workstation design. Environmental working conditions such as air quality and temperature, inadequate lighting, and noise are significant sources of distraction and discomfort affecting office workers’ physical and mental health (12). Inadequate air temperature, poor lighting, or glare as well as noise related to conversations, telephone calls, and notifications cause distractions and psychological distress that relate to musculoskeletal discomfort in office workers. Similarly, an association of poor lighting and air quality (21, 24, 25) with the occurrence of MSDs in teleworkers has been observed in several selected studies. Matsugaki et al. (24) showed that teleworkers are generally satisfied with their environmental conditions at home with only 16% of them reporting poor lighting and 25% reporting poor air quality. However, more than 77% of queried teleworkers reported that they have a dedicated place to work, which may relate to perceived positive experiences regarding environmental working conditions. Similarly, Montreuil and Lippel (14) have previously reported that teleworkers are more satisfied with domestic working conditions where they might experience more control over air quality and noise compared to office work, but only the ones having a dedicated place to work.

Lack of a dedicated place for work (11, 22, 24, 25) as well as experiencing frequent interruptions (19) by surrounding household noise or other household members (21, 26) are important psychosocial factors associated with MSDs in teleworkers. Frequent distractions and low perceived privacy (27) observed in teleworkers cause psychological distress and affect musculoskeletal health. Research suggests that women, in particular, may experience increased psychosocial demands due to multiple household roles and may be at an increased risk of negative mental and physical health outcomes while teleworking (36). A significant association has been observed between being a female and reporting MSD while teleworking (11, 20, 28–30). Women seem to be more frequently affected by work-related MSDs when compared to men regardless of occupation and work setting (37–39). Although reasons for such findings are yet unknown, previous research suggests that work and household demands as well as work-related physical and psychological demands may have a role in the greater prevalence of musculoskeletal discomfort in women (1). Paradoxically, women seem to perceive a greater value in teleworking when compared to men, seeing it as an opportunity for better work-family balance (40, 41). However, long working hours and high job demand frequently imposed on teleworkers may have the complete opposite effect on work-family balance. “In exchange” for greater flexibility in scheduling working hours, employers may raise expectations regarding employees’ workload and availability (42, 43) blurring the boundaries between work time and family time resulting in work–family conflict (27). Studies have shown that setting spatial and temporal boundaries between work time and family time activities as much as possible may be beneficial for work-family balance and teleworkers’ mental health in general (44).

Studies have shown that the pandemic brought an additional workload on teleworkers. Snodgrass et al. observed an increase in the frequency of teleworking in part-time teleworkers during the pandemic from an average of 28% to 48% of total working time (18) with increasing daily working hours as well (20, 28). Du et al. observed that despite contracted 7 h working days, workers spent an additional hour and a half working during the pandemic confinement (20). Reasons for increased working hours may arise from perceived job insecurity commonly reported in teleworkers during the pandemic (17) making them work harder to meet increased job demands, as well as in more frequent distractions due to an increased number of household members starting their work or school remotely. Several selected studies showed increased working hours as well as the frequency of teleworking to be associated with reported MSDs (11, 19, 20, 24, 25, 28, 31) probably due to physical and mental overload arising from ergonomic and psychosocial factors of telework.

Psychosocial, ergonomic, and environmental risks arising from telework may furtherly be worsened by observed changes in levels of physical activity and time spent in sedentary behavior during the pandemic (17). The aforementioned changes may be attributed to the nature of telework as well as pandemic-related confinement. Home-based work enables work without the usual office interruptions with less need to stand up and less mobility than within the company causing prolonged bouts of sitting behavior (17). Research suggests that home-based workers spend longer engaging in single bouts of sitting behavior when compared to office workers (45, 46). Additionally, high working demands and long working hours associated with telework may furtherly increase sedentary time altogether. On the other hand, by imposing lockdowns, the pandemic affected both work-related and leisure-time physical activity. Time spent in healthy ways of commuting such as walking or cycling has decreased (30). Leisure-time activities shifted from aerobic activities predominately performed outdoors (running, cycling, walking, swimming) toward strength and flexibility activities that can easily be performed at home (30). A systematic review analyzing changes in physical activity and sitting behavior during the COVID-19 pandemic reports a 5%–11% decrease in physical activity and a 6–67% increase in sitting behavior (47) with almost 60% of people unable to meet the required 150 min/week of moderate physical activity and therefore meeting the criteria for physical inactivity (48). Lockdown stringency level, as suggested by Wilms et al. (47), may play a role in the magnitude of the aforementioned changes. Sitting behavior and physical inactivity have previously been related to numerous negative health outcomes (49) including the onset or augmentation of musculoskeletal pain in the working population (50). The observed increase in sedentary behavior and decrease in physical activity in teleworkers is associated with musculoskeletal pain in several studies (11, 17, 21, 28), but not all (30, 51) implicating the need for further investigation of work-related and leisure-time physical activity in etiology and augmentation of chronic musculoskeletal pain.

## Conclusion

For a certain part of workers, telework will remain a common way of work in the post-pandemic period. Therefore, it is essential to identify telework-related risk factors for musculoskeletal disorders and address them with timely preventive measures tailored to each remote workstation’s risks and individual workers’ needs. Risk assessment of hazardous working conditions may be the first step in addressing risk factors for MSDs in teleworkers. However, resources enabling risk assessment in an organizational setting may not be always available in a remote setting making it difficult for employers to control teleworkers’ working conditions. For example, direct measurements and observational methods commonly used to assess biomechanical loads arising from working postures and repetitive movements in on-site workers are hardly applicable to home-based workers. Targeted checklists and questionnaires may be beneficial in the initial recognition of ergonomic hazards that may lead to increased biomechanical loads in remote setting (52). Quantification of biomechanical workloads in more advanced setting may be performed using wearable devices incorporating inertial sensing technology (53). In addition to risk assessment, organizational support in terms of equipment and education of teleworkers in preparing ergonomically suitable workstations as well as in taking regular breaks and reducing sitting time is essential (21, 54). Psychosocial risk assessment in remote settings may be performed using standardized tools commonly used in an on-site setting while taking into account specific telework-related psychosocial risks as well as specificities related to information and communication technology (55). To prevent psychosocial risks arising from high job demands and blurred work-family boundaries organizations should actively include teleworkers in decision-making regarding job requirements and deadlines (56). Teleworkers, on the other hand, may benefit from setting different forms of boundaries between work and family time to decrease distractions and maintain a good work-family balance.

## Author contributions

MM, HK, KB-K, and MB contributed to the conception and design of the study. JN and PJ performed the databases search. LB, BB, JČ, and MC selected and organized the relevant articles. MM, HK, and KB-K wrote the first draft of the manuscript. HK, MM, KB-K, MB, JN, and PJ wrote sections of the manuscript. All authors contributed to the article and approved the submitted version.

## Conflict of interest

The authors declare that the research was conducted in the absence of any commercial or financial relationships that could be construed as a potential conflict of interest.

## Publisher’s note

All claims expressed in this article are solely those of the authors and do not necessarily represent those of their affiliated organizations, or those of the publisher, the editors and the reviewers. Any product that may be evaluated in this article, or claim that may be made by its manufacturer, is not guaranteed or endorsed by the publisher.
